# Deletion 21q22.3 and duplication 7q35q36.3 in a Colombian girl: a case report

**DOI:** 10.1186/s13256-016-0988-2

**Published:** 2016-07-27

**Authors:** Felipe Ruiz-Botero, Harry Pachajoa

**Affiliations:** Faculty of Health, Universidad Icesi, Research Centre on Congenital Anomalies and Rare Diseases (CIACER), Calle 18 No. 122-135, bloque L, Oficina: 5025A Pance, Cali, Colombia

**Keywords:** 21q deletion, 7q duplication, Comparative genomic hybridization, Intellectual disability

## Abstract

**Background:**

Genetic disorders are a major cause in the etiology of cases with intellectual disability; however, analysis by a conventional technique such as cytogenetic karyotyping only allows the detection of chromosomal alterations in approximately 9.5 % of cases. The inclusion of new technologies such as high resolution microarray analysis has allowed the study of alterations in chromosomal segments that are less than 5 Mb in length; this has led to an increase in the diagnosis of these patients of up to 25 %.

**Case presentation:**

We report the first case of an 8-year-old Colombian girl of mixed race ancestry (Mestizo), with clinical features that include: delayed psychomotor and language development, intellectual disability, upward slanting palpebral fissures, divergent strabismus, low-set and rotated ears, tall and broad nasal bridge, flat philtrum, bifid uvula, posterior cleft palate, increased anteroposterior diameter of her chest, congenital heart defect type interventricular communication, scoliosis, and umbilical hernia. Genetic analysis was performed using comparative genomic hybridization array, which evidenced the deletion of a region of approximately 3.608 Mb on chromosome 21q22.3, and a duplication of 12.326 Mb on chromosome 7q35q36.3, these alterations affect approximately 112 and 186 genes, respectively.

**Conclusions:**

To date, this is the first report of an associated terminal deletion of 21q and 7q duplication in a patient with delayed psychomotor development and intellectual disability. We consider that future implementation of exome and RNA sequencing techniques, and analysis of their proteomic expression in a clinical context could lead to better analysis and interpretation of the genotype–phenotype correlation in cases similar to that described.

## Background

Genetic disorders are a major cause in the etiology of cases with intellectual disability; however, analysis by a conventional technique such as cytogenetic karyotyping only allows the detection of chromosomal alterations in approximately 9.5 % of cases [1]. The inclusion of new technologies, such as high resolution microarray comparative genomic hybridization (CGH) analysis, has allowed the study of alterations in chromosomal segments of lengths less than 5 Mb, allowing an increase in the diagnosis of up to 25 % of cases previously considered idiopathic syndromic [2–4].

Chromosomal constitutional imbalances are frequently associated with learning disabilities, impairment, dysmorphism, congenital anomalies, and impaired growth [5]. Chromosomal aneuploidies, particularly those involving chromosome 21, are one of the most prevalent human chromosomal alterations, happening in approximately 1 of every 700 births. Of these, a low percentage is due to partial trisomy or monosomies, which are difficult to detect because only a region of chromosome 21 is affected [6].

Chromosome 21 monosomy is a rare abnormality, especially when compared with the frequency of appearance of trisomy of chromosome 21 [7]. Very often fetuses with complete monosomy 21 die before or shortly after birth [8]; by contrast, cases with partial deletion of chromosome 21, which occur more often, have better survival expectancy and are heterogeneous regarding their phenotypic severity [9].

Although duplications of the long arm of chromosome 7 are well known, these are rare and their presence usually results in the apparition of multiple congenital abnormalities and cognitive deficits. Clinical features depend on the size of the duplicated segment and the possible presence of concomitant chromosomal deletions, secondary to rearrangements of chromosomal segments [10, 11].

We present the first report of a Colombian patient with 21q22.3 deletion and concomitant 7q35q36.3 duplication detected by CGH array and associated with dysmorphism, delayed development, cognitive deficit, and complex congenital heart disease.

## Case presentation

We report the case of an 8-year-old girl of Colombian origin and mixed race ancestry (Mestizo) who is the product of the first pregnancy of non-consanguineous parents; her mother was 32-years old and her father was 31-years old at the time of her gestation. Her mother’s prenatal history shows no evidence of teratogen exposure or any other relevant exposures or pathologies. Ultrasound reports during weeks 14 and 25 of gestation showed no morphological alterations.

Delivery care was performed at 38 weeks of gestation by spontaneous vaginal delivery, after which bilateral clubfoot and heart murmur were identified. An echocardiogram showed the presence of an atrial septal defect of 7.4 mm with a left to right shunt, dilated coronary sinus, subaortic interventricular communication of 4.8 mm and 6.8 mm with a left to right shunt. She underwent surgery for her congenital heart disease at 51-days old due to the presence of dyspnea, fatigue, anorexia, and diaphoresis. Currently she has a unicameral pacemaker.

**Fig. 1:**
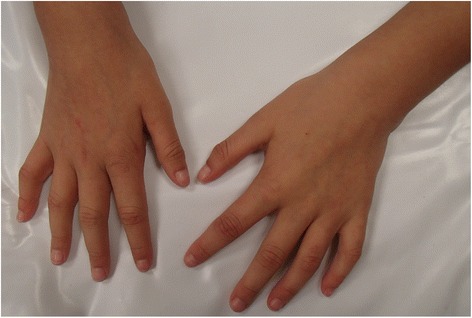
Image of the patient at 7 years of age. Note bilateral fifth finger clinodactyly

**Fig. 2:**
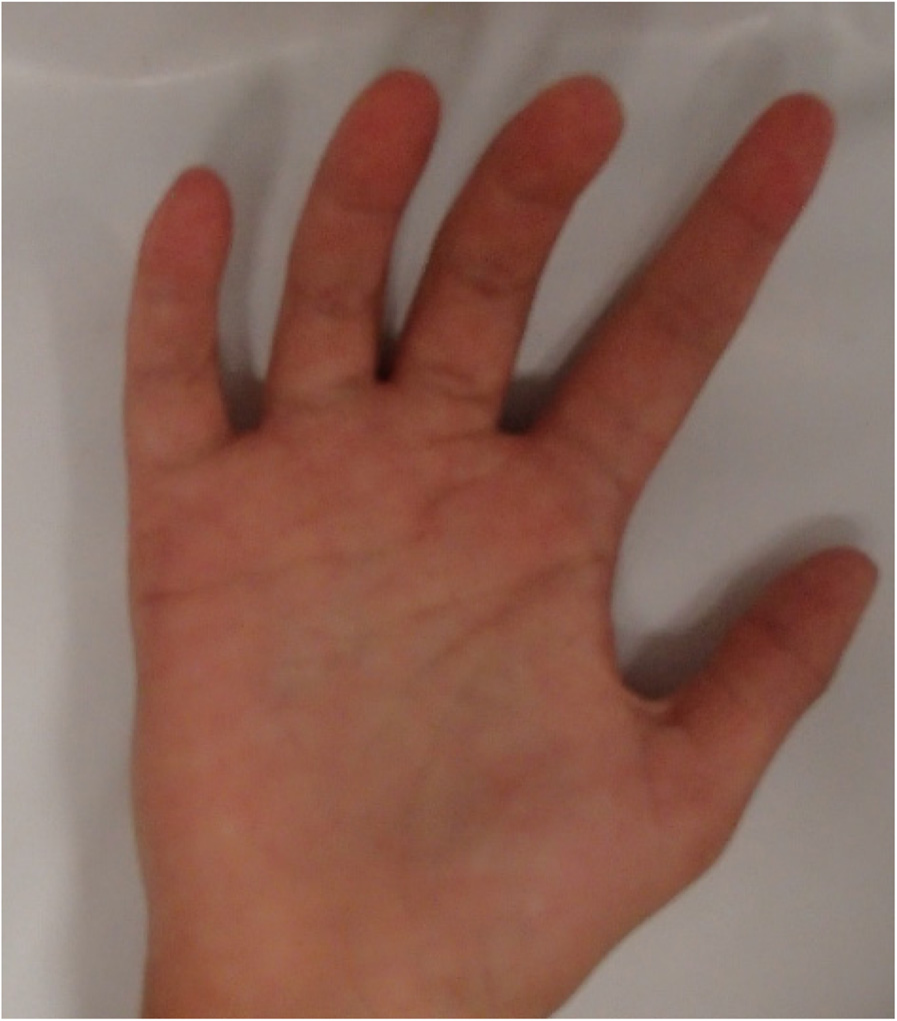
Image of the patient at 7 years of age. Note right thumb with presence of two interphalangeal creases and thenar hypoplasia

She is currently 8 years of age, and has the following clinical findings: delayed psychomotor and language development, cognitive deficits, anthropometric measurements within normal percentiles, upward slanting palpebral fissures, divergent strabismus, rotated and low-set ears, tall and broad nasal bridge, flat philtrum, bifid uvula, posterior cleft palate, increased anteroposterior diameter of the thorax, scoliosis, and umbilical hernia. Her extremities evidenced clinodactyly of her fifth bilateral fingertips, bilateral ulnar deviation, right thumb with the presence of two interphalangeal grooves, thenar hypoplasia, gait abnormality, bilateral clubfoot (corrected), bilateral sandal gap, nail split of the second toe of her right foot, and a social personality (see Figs. 1 and 2).

**Fig. 3:**
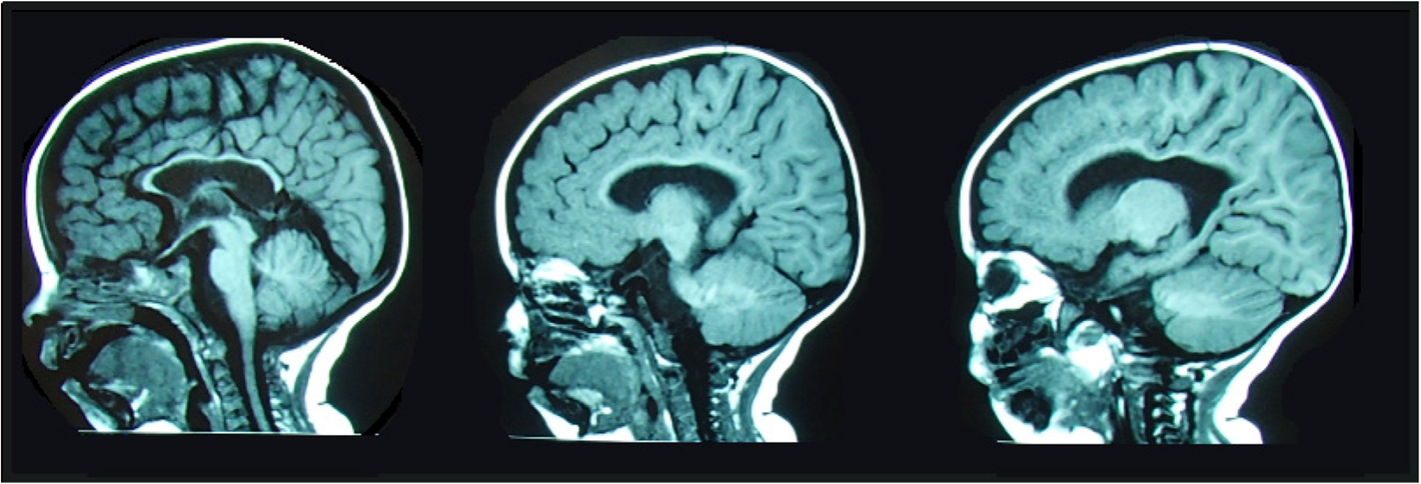
Magnetic resonance imaging of the brain evidencing corpus callosum hypoplasia

Complementary studies were conducted: magnetic resonance imaging (MRI) of her brain evidenced brain stem and corpus callosum hypoplasia, and cortico-subcortical atrophy; thoracic spine MRI showed a left middle thoracic scoliotic curve (see Fig. 3). Electroencephalogram had an abnormal vigil tracing with frequent repetitive discharge points, isolated biphasic spike, associated with right frontotemporal high voltage slow waves. Hip radiography confirmed bilateral coxa valga and hip subluxation; foot and ankle radiography documented a varus foot, her first metatarsal talus mechanical axis was altered by lateralization of the astragal axis, and the presence of ankle valgus.

**Fig. 4:**
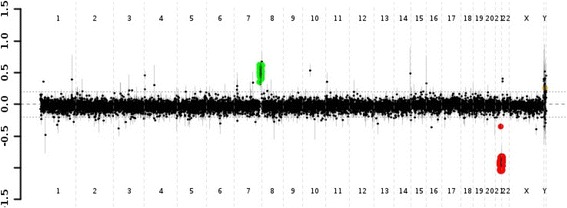
Comparative genomic hybridization array result evidencing in *green* copy number gain of 12.326 Mb on chromosome 7q35q36.3, and in *red* loss of 3.608 Mb on chromosome 21q22.3

G-banding karyotyping was performed in 20 metaphases, which reported a 46,XX female; subsequently, chromosomal analysis was performed by CGH array that showed a loss of approximately 3.608 Mb on chromosome 21q22.3, and a copy number gain of 12.326 Mb on chromosome 7q35q36.3 (see Fig. 4); these alterations affect approximately 112 and 186 genes, respectively (see Table 1). Both copy number changes are terminal, which suggests an unbalanced translocation between chromosomes 7 and 21, resulting in a derivative chromosome 21.

**Table 1:** Reference of 50 affected genes by RefSeq database for altered regions on chromosomes 7 and 21

Chromosome	MIN START hg19	MAX START hg19	MIN STOP hg19	MAX STOP hg19	RefSeq.(50 max.)
21	44482517	44482408	48090317	48157577	CBS, U2AF1, CRYAA, SIK1, NCRNA00319, NCRNA00313, HSF2BP, RRP1B, PDXK, CSTB, RRP1, LOC284837, AGPAT3, TRAPPC10, PWP2, C21orf33, ICOSLG, DNMT3L, AIRE, PFKL, C21orf2, TRPM2, LRRC3, TSPEAR, C21orf90, KRTAP10-1, KRTAP10-2, KRTAP10-3, KRTAP10-4, KRTAP10-5, KRTAP10-6, KRTAP10-7, KRTAP10-8, KRTAP10-9, KRTAP10-10, KRTAP10-11, KRTAP12-4, KRTAP12-3, KRTAP12-2, KRTAP12-1, KRTAP10-12, UBE2G2, SUMO3, PTTG1IP, ITGB2, LOC100505746, C21orf67, C21orf70, NCRNA00163, NCRNA00162.
7	146762651	146752081	159088636	159321559	CNTNAP2, MIR548I4, MIR548F4, MIR548F3, MIR548T, C7orf33, CUL1, EZH2, PDIA4, ZNF786, ZNF425, ZNF398, ZNF282, ZNF212, ZNF783, LOC155060, ZNF777, ZNF746, ZNF767, KRBA1, ZNF467, SSPO, ZNF862, LOC401431, ATP6V0E2, ACTR3C, LRRC61, C7orf29, RARRES2, REPIN1, ZNF775, LOC728743, LOC285972, GIMAP8, GIMAP7, GIMAP4, GIMAP6, GIMAP2, GIMAP1, GIMAP1-GIMAP5, GIMAP5, LOC100128542, TMEM176B, TMEM176A, ABP1, KCNH2, NOS3, ATG9B, ABCB8, ACCN3.

*MAX* maximum, *MIN* minimum

Complementary studies were performed with both parents using fluorescence *in situ* hybridization (FISH). The results showed our patient’s mother has a reciprocal translocation between chromosomes 7 and 21 ish t(7;21)(q35;q22.3) (RP11-10L20-, RP11-75J5 +; RP11-75J5-, RP11-10L20 +).

## Discussion

We report the case of a Colombian girl with a deletion on chromosome 21q22.3 and duplication on chromosome 7q35q36.3, secondary to the presence of her mother’s reciprocal translocation between chromosomes 7 and 21. This is the first case reported in the literature in which both mutations occur simultaneously.

Partial deletion of chromosome 21q is a rare condition, with over 45 cases reported in the literature [9, 12–16]. Their phenotype is widely heterogeneous; Lyle *et al*. [14] reported 11 cases of partial monosomy of chromosome 21, differentiating three affected regions that are associated with the severity of the expressed phenotype varying from moderate, severe, to lethal. These regions are: Region one comprises deletion of centromere to 32.3 Mb (hg19), region two from 32.2 Mb to 37.1 Mb (hg19), and region three from 37 Mb to the telomere. The present case report has a deletion which comprises region three, which was reported by Roberson *et al*. as the most commonly altered region in these cases and with the least severe phenotypic expression [14, 16].

Lyle *et al*. [14] performed a correlation between genotype and phenotype in 30 patients with partial trisomy of chromosome 21 and 11 patients with partial monosomy 21. Of the cases described by Lyle *et al*. [14] one patient was identified with deletion 21q22.3 which had a mild phenotype that included hypertelorism, epicanthic fold, downward slanting palpebral fissures, long nose, seizures, and marfanoid habitus; such findings are not evidenced in our current case report. However, when compared with the phenotypic characteristics in other patients with partial monosomy 21, common characteristics such as short neck line and low hairline, cleft palate, broad nasal bridge, broad mouth, cardiac abnormalities, clinodactyly, hypotonia, hypermobility, and cognitive deficits are evidenced (see Table 2) [14].

**Table 2:** Phenotype comparison of patients with partial monosomy of the 21q

Clinical features	1	2	3	4	5	6	7	8	9	10	11	Actual report
Short stature	+	+				+			–		+	–
Short neck	+	+							–			+
Microcephaly	–	+					+		–		+	–
Brachycephaly									–			–
Dolichocephaly						+			–			–
Low hairline		+							–			+
Epicanthic eye fold	–	–				+			+		–	–
Hypertelorism	–					+			+		+	–
Microphthalmia	+								–		–	–
Highly arched palate	+	+									–	+
Down-slanted palpebral fissures	–	–							+		+	–
Symbrachydactyly	+						+		–			–
Brushfield spots									–			–
Flat nasal bridge									–		–	–
Broad nasal bridge	+	+							–		–	+
Large nose	+	+							+		–	–
Pronounced median raphe of the philtrum	+								–			–
Broad mouth	+	+							–		+	+
Large ears	+	+							–		+	–
Cardiac anomaly	–	–							–		+	+
Clinodactyly of fifth finger	–	+							–		+	+
Palmar crease	–	+							–			–
Hypotonia		+				–			–			+
Hypertonia	+	+				–			–			+
Seizure							+		+		–	
Ligamentous laxity									–		–	+
Intelligence quotient or mental retardation	+	+	+			+	+		+		+	+

Original table taken from Lyle *et al*. [14] and modified for this article by the authors; patients labeled 1 to 11 are patients 31 to 42 in the original report

Likewise, features not described by Lyle *et al*. [14] were also identified such as club foot and cleft palate; nevertheless, these features are described in the phenotypic spectrum 7q duplication [11, 14].

The 21q deletion has been related to alterations in the morphogenesis of the human brain, dosage-sensitive genes in this region contribute to cortical development, and deletions in this region have been associated with the presence of cortical dysplasia, including colpocephaly and hypoplastic corpus callosum. Our patient does not show colpocephaly, this indicates that the telomeric region of 21q22.3 identified in her is not related to colpocephaly, and the responsible region for colpocephaly could be narrowed to region 21q22.1q22.3 [12, 13, 17].

The duplication of the long arm of chromosome 7 is a rare pathology, of which approximately 54 cases are described in the literature; the majority of them are inherited from a balanced chromosomal rearrangement of a parent [11, 18–21]. The partial 7q duplications have been classified into four groups according to the affected region [21]. This classification is considered artificial due to the frequent lack of specificity of the clinical findings in relation to other genetic syndromes [21].

Group one includes those patients with complete duplication of the long arm, whose clinical features include: hypertelorism, low-set ears, micrognathia, short neck, abnormalities of the genitourinary tract, and early death [20, 21].

Group two includes duplications of large segments, starting in proximal bands until reaching the telomere. The clinical expression and severity is highly variable; the most common findings comprise cleft palate, microretrognathia, and urinary tract abnormalities [20, 21].

Group three is made up of those cases with interstitial duplications of variable size. Between the reported cases a proximal break point located between 7q21 and 7q22 is commonly referenced. The common features described in this group include low birth weight, macrocephaly, prominent forehead, hypertelorism, low-set ears, and short neck [20, 21].

Group four includes distal duplications, such as those in the case described in this case report. The most common phenotypic features are: macrocephaly, prominent forehead, small nose, low-set ears, and developmental delays (usually severe) [20, 21].

Scelsa *et al*. [21] presented a comparative table of the clinical features described in previous reports of 7q duplication [21]. When comparing the characteristics of these cases with our current case report, there are several consistent features such as: cleft palate, low-set ears, short neck, congenital heart disease, strabismus, psychomotor retardation, hypotonia, and club foot (see Table 3) [21].

**Table 3:** Phenotype comparison of patients with distal duplication 7q

Anomalies		Rodriguez et al. q31.2-qter [22]	Scelsa et al. q32-qter [21]	Bartsch et al. q33-qter [23]	Bartsch et al. q33-qter [23]	Romain et al. q34-qter [24]	Romain et al. q34-qter [24]	Verma et al. q36-qter [25]	Present report q35-q36.3
Early death					Abortion				
Low birth weight			+		Fetus	+	+		
Major anomalies	Congenital heart defects		+						+
	Genital-urinary defects	+	+		+				
	Skeletal anomalies	+			+	+	+		
	Strabismus						+		+
	Failure to thrive	+	+	+			+		
	Developmental delay	Severe	Severe	Severe		Moderate	Severe	Moderate	Severe
	Club feet								+
	Hypotonia		+	+			+		+
Minor facial anomalies	Macrocephaly	+	+	+		+		+	
	Frontal bossing	+	+	+	+			+	
	Hypertelorism	+	+				+		
	Narrow palpebral fissures		+						
	Epicanthus			+			+		
	Down-slanting eyes		+	+					
	Small nose		+	+	+	+	+		
	Depressed nasal bridge	+	+	+	+		+		
	Micrognathia/Cleft palate	+	+	+	+				+
	Malformed ears		+						
	Low-set ears	+	+	+	+	+	+		+
	Short neck	+	+	+	+		+		+

Original taken from Scelsa *et al*. [21] and modified for this article by the authors

It is worth noting that the 21q deletion may present among its phenotypic findings a short neck, hypotonia, and cardiac abnormalities as does the 7q duplication; these traits are evident in our patient, making it difficult to correlate the individual influence of each of these genotypic abnormalities with the phenotypic expression of the patient. This difficulty is increased when taking into account the phenotypic heterogeneity described for each case.

## Conclusions

To date, this is the first report of the concomitant presence of a terminal 21q deletion and 7q duplication in a patient with delayed psychomotor development and cognitive deficit. Despite the difficulty of excluding the mutual effect of both genotypic alterations on the patient’s phenotype, we consider that the present analysis will help to determine a better phenotype–genotype correlation analysis in cases with partial monosomy 21 and/or duplication 7q.

We considered that implementation of sequencing techniques such as exome sequencing, RNA sequencing, and use of proteomics expression analysis in a clinical context could lead to better analysis and interpretation of the phenotype–genotype correlation of similar cases. However, the current use of CGH array, particularly in developing countries, allows a proper analysis of chromosomal imbalances and an appropriate correlation between the etiology and clinical-pathological expression; hence, it becomes a vital tool to aid clinicians in the diagnosis and treatment of such pathologies.
